# Enzyme (Single and Multiple) and Nanozyme Biosensors: Recent Developments and Their Novel Applications in the Water-Food-Health Nexus

**DOI:** 10.3390/bios11110410

**Published:** 2021-10-21

**Authors:** Lynette Alvarado-Ramírez, Magdalena Rostro-Alanis, José Rodríguez-Rodríguez, Juan Eduardo Sosa-Hernández, Elda M. Melchor-Martínez, Hafiz M. N. Iqbal, Roberto Parra-Saldívar

**Affiliations:** Tecnologico de Monterrey, School of Engineering and Sciences, Monterrey 64849, Mexico; A00814259@itesm.mx (L.A.-R.); magda.rostro@tec.mx (M.R.-A.); jrr@tec.mx (J.R.-R.); eduardo.sosa@tec.mx (J.E.S.-H.); elda.melchor@tec.mx (E.M.M.-M.); hafiz.iqbal@tec.mx (H.M.N.I.)

**Keywords:** biosensors, enzymes, nanozymes, laccase, glucose oxidase, horseradish peroxidase

## Abstract

The use of sensors in critical areas for human development such as water, food, and health has increased in recent decades. When the sensor uses biological recognition, it is known as a biosensor. Nowadays, the development of biosensors has been increased due to the need for reliable, fast, and sensitive techniques for the detection of multiple analytes. In recent years, with the advancement in nanotechnology within biocatalysis, enzyme-based biosensors have been emerging as reliable, sensitive, and selectively tools. A wide variety of enzyme biosensors has been developed by detecting multiple analytes. In this way, together with technological advances in areas such as biotechnology and materials sciences, different modalities of biosensors have been developed, such as bi-enzymatic biosensors and nanozyme biosensors. Furthermore, the use of more than one enzyme within the same detection system leads to bi-enzymatic biosensors or multi-enzyme sensors. The development and synthesis of new materials with enzyme-like properties have been growing, giving rise to nanozymes, considered a promising tool in the biosensor field due to their multiple advantages. In this review, general views and a comparison describing the advantages and disadvantages of each enzyme-based biosensor modality, their possible trends and the principal reported applications will be presented.

## 1. Introduction

Biosensing is the detection signal of interaction between biological molecules with other molecules or analytes, and the device employed to sensing these interactions is a biosensor [1]. Nowadays, the rapid analysis and detection of different analytes play an essential role. Traditional chemistry analysis could represent a long time to detect because it requires extensive and expensive devices impeding the quick response. The biosensor’s objective is to provide a fast and accurate response about a particular analyte with operational simplicity [2,3,4]. Additionally, it is possible to detect the analytes cost-effectively without the need for complicated and expensive sample preparation. Since they can be miniaturized, they could be considered for portable in situ analysis, which is critical for point-of-care diagnosis [5]. Biosensors represent a reliable alternative that can be applied to several analytical processes in numerous fields. They are successfully implemented for early disease identification, toxins, viruses, elevated blood levels, etc. [6,7,8,9,10]. In the food industry, biosensors are used to detect food allergens, food contamination, or antioxidant power [11,12,13]. In the environment, biosensors could detect pollution in the air or contaminants in water or soil [14,15].

Enzyme-based biosensors are extensively used for their high selectivity and sensitivity. For example, reduction-oxidation enzymes have been extensively used in enzyme-based biosensors due to their ability to catalyze reactions based on electron transfer. The most used enzymes are glucose oxidase and horseradish peroxidase. Additionally, laccase is an emerging enzyme with the capacity to oxidase a broad range of substrates without H_2_O_2_ in the reaction medium. Thus, electrodes could achieve the recognition of multiple analytes with two or more enzymes. However, long-term stability could be challenging due to the instability of enzymes [16]. Thus, immobilization is essential to stabilize enzymes and increase their reusability. Even when research has been focused in increased the stability of enzymes, they have limited chemical and biological stability and high cost due to their purification process. Artificial enzymes are also known as nanozymes, are nanomaterials with enzyme-like activity. They have high stability and represent an excellent choice due to their simple preparation technologies. An essential advantage of nanozymes is that to can change their catalytic activity by varying shape, structure, and composition [17]. To date, a significant number of nanozyme-based biosensors have been reported. 

This review mainly focuses on the related biosensor technology, specifically in the enzyme-based biosensor and nanozyme-based biosensors. Their recent developments and variants focus on three principal enzymes: horseradish peroxidase, glucose oxidase, and laccase, as well as nanozymes with oxidase, peroxidase, and laccase-like activity. Finally, a comparison between these systems and their applications will be presented.

## 2. Biosensors

**Figure 1:**
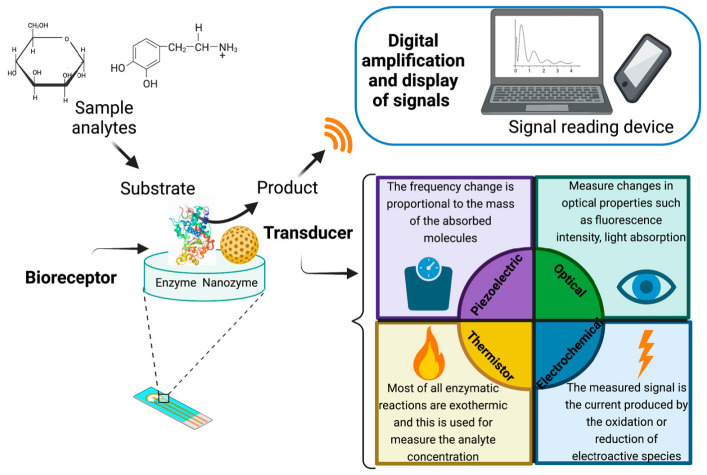
Main biosensing elements and their mechanistic role.

Three main parts constitute a biosensor: a recognition element or bioreceptor, that is a biomolecule that identifies the analyte; a transducer, which converts the signal (biological/chemical) into a detectable signal; and a signal reading device, which measures the transducer signal (Figure 1) [12,18,19]. The bioreceptor is the most crucial aspect of biosensors [20]; it is fundamentally any organic body that should detect an analyte from the medium of interest remaining other potential interfering species [1]. Different types of receptors have been reported as enzymes, microorganisms, nucleic acid fragments, antibody fragments, etc. [19]. The transducer can also be an electrochemical, optical, thermistor and piezoelectric [21]. An essential advantage in biosensors is that the bioreceptor and the transducer are integrated into one single sensor. This combination makes it possible to measure the analyte without reagents [2].

Different approaches can be used for their classification. Commonly, biosensors can be classified on the biological component used, the type of signal transduction they employed, and the type of detected analyte [21]. The following sections will be focused on the bioreceptor, specifically, the use of enzymes and other materials with similar characteristics, focusing on the enzyme-based biosensors (single and multiple) and nanozyme biosensors. 

## 3. Enzyme Based Biosensors

Enzyme-based biosensors were the earliest biosensors. In 1962, Clark proposed the idea of enzyme electrodes for a glucose sensor [22]. Subsequently, enzyme-based biosensors have been experimenting a massive growth in several applications [18]. The biological component used in enzyme-based biosensors is an enzyme. Enzymes are biological macromolecules with catalytic activity, high selectivity, and responsible for speeding up biochemical reactions under mild conditions [23]. These macromolecules can attach to one particular molecule or analyte, but not others to ensure the analyte selectivity. Due to their high specificity, simplicity, and scalability, enzyme-based biosensors represent a fast, precise, and continuous monitoring of analytes [24]. Additionally, the high specificity of enzymes enhances the ability to detect lower analyte concentration limits [18]. Additionally, the catalytic action can be influenced by the substrate concentration, temperature, pH, and inhibitor presence [18]. The enzyme functions could be the generation of electroactive species or an electroactive reactant’s consumption, causing the direct measurement of the analyte [25], or for oxidation or reduction of a molecule, which can be monitored electrochemically [26].

**Figure 2:**
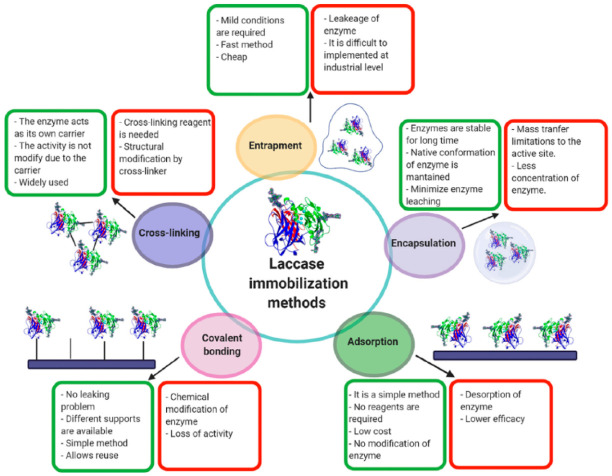
Schematic illustration of five basic immobilization techniques along with their advantages (**green**) and disadvantages (**red**). Reprinted from Ref. [29] with permission from Elsevier. License Number: 5151170420861.

The crucial factor in enzyme-based biosensors is the assembly or immobilization of the enzyme on the electrode surface [16]. If the immobilization is not correctly done, the accessibility of the active site, the stability through time, and the enzyme’s reusability could be affected. The enzymes can be immobilized on the transducer surface to improve the stability and reproducibility of the detection. The choice of support material is essential for conferring stability, selectivity and even improve enzyme activity. Consequently, the support material must be inert, stable, and resistant [27]. The immobilization technique is highly significant; without immobilization, the enzyme cannot be stable and reusable. The immobilized enzymes can be used continuously and can maintain their catalytic activity. Adsorption, covalent bonding, crosslinking, encapsulation and entrapment are the main methods used for immobilization [28,29]. In Figure 2 are represented the immobilization techniques with their advantages and disadvantages. The simplest methods are adsorption, encapsulation, and entrapment. Adsorption is inexpensive and straightforward; however, the enzymes have weak associations with the support [27]. Entrapment gives the enzyme high stability; however, the matrix can interfere with substrates’ diffusion to the enzyme’s active site. Covalent bonding is the most used method because a stable complex between the enzyme and support is generated [16]. Nevertheless, the formation of the covalent bonding could affect the enzyme activity. Crosslinking immobilization improves the stability and efficiency due to the stable binding between enzymes, generally formed with a reactant as glutaraldehyde. However, with reagents, conformational changes in the structure can affect the enzyme activity [21].

**Figure 3:**
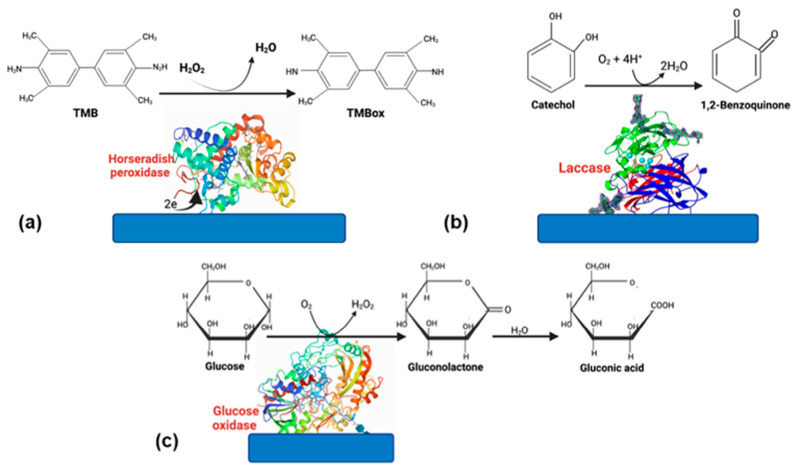
Enzymes commonly used in biosensors. (**a**) HRP, (**b**) laccase, and (**c**) glucose oxidase.

Even though the advantages of using enzymes, some disadvantages, such as the rapid loss of enzyme activity due to its interactions with the electrode surface, cause a biosensor’s lifespan is only 2–4 weeks. However, if the enzyme is well stabilized, this can increase [30]. Therefore, choosing a suitable matrix and an excellent strategy to immobilize the enzyme [29]. Enzyme-based biosensors have tremendous applications in food, medicine, and environmental monitoring. Oxidoreductases and peroxidases are the most-reported enzymes in biosensors because they are very stable catalyzing oxide reduction reactions [31]. In this section, we will focus on glucose oxidase (GOx), horseradish peroxidase (HPR), and laccase (Figure 3); these enzymes have been successfully used for different applications in biosensors. 

### 3.1. Horseradish Peroxidase

Horseradish peroxidase (HRP, EC 1.11.1.7) is an enzyme that belongs to the group of oxidoreductases. HRP is extensively distributed in nature, and its purification process is relatively simple. The principal source of extraction is the horseradish root. Some advantages reported are high activity and selectivity, resistance to inhibition by substances over a vast concentration, high operability, and reliability over a broad range of treatment conditions [32,33]. In addition, it can catalyze organic and inorganic substrates’ oxidation by reacting with H_2_O_2_ and similar molecules [34]. Therefore, HPR is has been widely used in biosensors for H_2_O_2_ determination [30]. 

The detection mechanism of the H_2_O_2_ biosensor depends on the electrode (modified or not) and whether the mediator is used or not. The mediator facilities the electron transfer between enzymes and electrodes. In the presence of a mediator, the H_2_O_2_ in the solution is reduced by the HRP. On the other hand, the mediator is oxidized in the enzymatic reaction by itself. Moreover, the oxidized mediator is finally reduced on the electrode, with a change in the current. If the mediator is not present, the enzyme is converted to its oxidized form after being reduced at the electrode surface by direct electron transfer [35].

Hydrogen peroxide is an essential intermediate in enzymatic reactions. The detection of H_2_O_2_ is essential in medicine, food, and environmental assays (Table 1). As a result, the development of biosensors for H_2_O_2_ detection has been extensive [30]. Different materials have been reported to improve the response between the electrode and the HRP enzyme, for example, gold nanoparticles (AuNPs), cadmium sulfide, nanofibers, carbon nanomaterials. Carbon nanomaterials have unique properties, such as good biocompatibility, fast electron transfer, and excellent mechanical flexibility. Feizabadi et al. [30] immobilized HRP on a modified multi-walled carbon nanotube by γ- aminobutyric acid (GABA) on a glassy carbon electrode. The covalent bonding formed between the enzyme and GABA increased the stability and reproducibility of the biosensor. Due to its characteristics as low detection (0.13 µM) and extensive linear range (0.2 to 281 µM for H_2_O_2_), the biosensor could be used to quantify H_2_O_2_ in human plasma. 

Da Silva Freires et al. [36] developed a biosensor based on copper (I) sulfide (Cu_2_S) and HRP immobilized on a fluorine-doped tin oxide modified glass slide (FTO) for the determination of 1,4-dihydroxybenzene (DHB). The biosensor showed good selectivity for DHB, and good accuracy when DHB was determined in skin cream samples, presenting recovery percentages for the analyte in the samples between 99.89 and 100.70%, suggesting a good accuracy of the proposed method.

For cancer detection, exosomes can be an alternative as it is a non-invasive technique. Additionally, it is desirable for a cost-effective and instance detection in clinical diagnosis. Zeng et al. [37] developed a versatile biosensor to detect cancer-derived exomes (HepG2 cell-derived). HRP encapsulated DNA nanoflowers were the recognition elements and signal generation. A change of color was proportional to the concentration of exosomes. The system showed a satisfactory colorimetric response toward target exosomes within the working range from 5.0 × 103 to 5.0 × 106 particles/μL at a low detection limit of 3.32 × 103 particles/μL. López-Marco et al. [38] used HRP in a 3D-printed graphene/polylactic electrode and compared the detection of H_2_O_2_. The AuNPs facilitate and enhance electron transfer. However, they found that biosensors without AuNPs displayed better stability over time. The response of biosensors was evaluated in human serum. 

**Table 1:** Horseradish peroxidase biosensors.

Material	Transduction System	Application		Linear Range with a lineal Correlation	Limit of Detection (LOD)	Ref.
Glass plate covered with fluorine-doped tin oxide (FTO)Copper (I) sulfide (Cu_2_S) and fluorine-doped tin oxide modified glass slide	Photoelectrochemical	Health	1,4-dihydroxybenzene (DHB)	10 nmol L^−1^ up to 1 mmol L^−1^ (R = 0.998)	4.0 nmol L^−1^	[36]
Encapsulated DNA nanoflowers of magnesium pyrophosphate crystals	Colorimetric	Health	Rapid screening of cancer-derived exosomes	5.0 × 10^3^ to 5.0 × 10^6^ particles/μL (R^2^ = 0.9846)	3.32 × 10^3^ particles/μL	[37]
Polydimethylsiloxane (PDMS) deposited into a polystyrene tube	Chemiluminescent	Health	Quantification of H_2_O_2_ as the oxidizing agent	0.06−10 μM (R^2^ = 0.999)	0.02 μM	[4]
Modified multi walled carbon nanotube by γ-aminobutyric acid	Electrochemical	Food, health, environmental	Detection of hydrogen peroxide	2.0 × 10^−7^ M to 2.81 × 10^−4^ M (R^2^ = 0.998)	0.13 μM	[30]
3D-printed graphene/polylactic (PLA) electrode with gold nanoparticles	Electrochemical	Environmental and biomedical fields.	Hydrogen peroxide detection	25–100 µM (R = 0.996)	11.1 µM	[38]
HRP-encapsulated protein nanoparticles in an Au electrode surface	Electrochemical	Clinical applications	Hydrogen peroxide detection	0.01–100 μM	0.01 µM	[39]
Modified platinum electrode covered with poly(4,7-bis(5-bromothiophen-2-yl) benzothiadiazole)	Electrochemical	Health	17β- estradiol	0.1 to 200 mM (R^2^ = 0.99)	105 nM	[40]
Tungsten microwire modified with AuNPs and 3-mercaptopropionic acid	Electrochemical	Health	Determination of hydrogen peroxide	5 nM to 5 µM (R = 0.999)	800 pM	[41]
Modified acrylic microspheres	Electrochemical	Food	Chilli hotness determination	0.75–24.94 μM (R^2^ = 0.992)	0.39 µM	[42]

### 3.2. Glucose Oxidase

Nowadays, measuring blood glucose has been widely studied due to its relevance for health care [8]. Glucose oxidase (GOx) is the principal enzyme used for the devices of glucose monitoring. It is a glycoprotein that possesses orthophosphates proteins. This enzyme has unique properties such as dispersibility in water, resistance to precipitation, and stability. All these advantages make GOx efficient for glucose monitoring in blood or saliva [8]. The continuous monitoring of glucose is vital in diabetes mellitus disease. Therefore, the production of a simple, cost-effective, accurate, and rapid sensor is essential. GOx has still been used for glucose detection due to its reliable stability and substrate specificity. The principle of its operation is the enzymatic oxidation of glucose and after the electrochemical oxidation of H_2_O_2_ [43].

Electrochemical biosensors have been used to detect glucose using GOx (Table 2) [44]. The GOx enzyme converts the glucose to gluconic-d-lactone by reducing the flavin adenine dinucleotide (FAD) to FADH_2_. Then, H_2_O_2_ is produced due to FADH2 deoxidized by dissolved O_2_. Subsequently, the H_2_O_2_ is oxidized to O_2_ when a working potential is applied, and the electric current produced in the biosensor is proportionate to the glucose concentration [45]. The incorporation of nanomaterials as biosensor components has enhanced their performance. Bagyalakshmi et al. [44] prepared ZnO nanorods with chitosan. GOx was immobilized by the adsorption method. The ZnO nanorods were a successful platform for the immobilization of GOx due to their high surface area and displayed a good performance for displaying glucose. The use of carbon nanotubes in glucose biosensors have been improved the enzyme stability and specificity. Jayakumar et al. [46] reported an adsorbed osmium-based redox polymer crosslinked with GOx. It was possible to use less nanoconjugate due to the covalent bond between GOx and multiwalled carbon nanotubes (MWCNTs). Green approaches also have been made for the development of glucose biosensors. Yang et al. [9] developed an enzyme electrode based on AuNPs, PNE, and GOx for glucose detection by a green method. The biosensor presented high sensitivity to glucose and high response of fewer than 3 s. Redox mediator p-benzoquinone was added to enhance the linear detection range and sensitivity

### 3.3. Laccase

Laccase (benzenediol: oxidoreductase, E.C. 1.10.3.2) is a multi-copper oxidase considered a green catalyst due to combines the four-electron reduction of dioxygen to water with the one-electron oxidation of four substrate molecules [48,49]. Laccase can be produced by insects, plants, bacteria, or fungi. It is considered a suitable enzyme due to its excellent catalytic properties [50,51]. The substrate range is vast, and they can oxidase different compounds, resulting in the application of numerous biotechnological applications such as environment, food, and biosensors (Table 3) [52]. 

The laccase biosensor is of the third-generation type. Due to direct electron transfer between the electrode and enzyme, there is no need for a mediator. Laccases are immobilized on the electrode’s surface, and they are oxidized by oxygen and then are reduced by the substrate acting as electron donors for the oxidized form of the enzyme. A reduction current will be observed to reduce the products, which is proportional to their concentration. Unlike peroxidases, laccase-based biosensors only need oxygen and are already present in the solutions, so it is unnecessary to H_2_O_2_ for its catalysis [53]. 

Laccase biosensors have been used widely for dopamine detection. However, even when some analytes’ determination as dopamine has been studied extensively, novel techniques and approaches have been developed. Wardak et al. [10] constructed a laccase-based biosensor constructed by Soft Plasma Polymerization technique for dopamine detection. This technique enhances the sensitivity, and it was proved for pharmaceutical samples with satisfactory results. Furthermore, the use of polysaccharides has been explored. An exopolysaccharide (EPS) named botryosphaeran, and MWCNT were used to immobilize laccase on a glassy carbon electrode to detect dopamine. Even in the presence of other molecules as uric acid, the biosensor could determine the presence of dopamine [54]. Additionally, the fluorescence principle has been used for dopamine detection. Sangubotla and Kim [55] developed a fiber-optic biosensor with carbon dots and laccase. This material presents excellent features such as hydrophobicity, tunable photoluminescence, and biocompatibility.

**Table 3:** Laccase biosensors.

Material	Transduction System	Application		Linear Range with a Lineal Correlation	Limit of Detection	Ref.
Laccase hybrid microflowers synthesized with Cu_3_(PO_4_)_2_⋅3H_2_O	Optical	Health, clinical diagnosis application	Quantification of epinephrine	1–400 μM (R^2^ = 0.999)	0.6 μM	[56]
Carbon dots bio functionalized with 3-(aminopropyl)-triethoxysilane	Optical	Health. Clinical diagnosis application. Diagnosis of Alzheimer’s and Parkinson’s diseases.	Detection of dopamine	0–30 μM (R^2^ = 0.995)	41.2 nM	[55]
Multi-walled Carbon Nanotubes modified glassy carbon electrode	Electrochemical	Diagnosis of Alzheimer’s and Parkinson’s diseases.	Dopamine detection	0.1 μmol/dm^3^ to 10 μmol/dm^3^ and from 10 µmol/dm^3^ to 50 µmol/dm^3^	3.63 μA·dm^3^/μmol and 1.33 μA·dm^3^/μmol	[10]
Fe_3_O_4_@SiO_2_ microspheres stabilized onto glassy carbon electrode	Electrochemical	Health	Dopamine detection	1.5–75 μmol L^−1^ (R = 0.9980)	0.177 μmol L^−1^	[57]
Glassy carbon electrode layered with multi-walled carbon nanotubes using a film of botryosphaeran	Electrochemical	Health	Dopamine and spironolactone detection	2.99–38.5 μmol L^−1^ (R^2^ = 0.995)	0.127 μmol L^−1^	[54]
Carbon paper electrodes with layered two-dimensional molybdenum disulfide (MoS_2_) in flowers (MoS_2_-F) and ribbons (MoS_2_-R)	Electrochemical	Synthetic urine sample	Dopamine detection	0.1 to 0.5 µM and from 1 to 5 µM (R^2^ = 0.993)	10 nM	[58]
6,9-bis(4-hexylthiophen-2-yl)-11H- indeno[2,1-b]quinoxalin-11-one (M1)) polymerized on electrode surface.	Electrochemical	Environmental applications	Catechol in water	005–0.175 mM (R^2^ = 0.994)	9.86 μM	[59]
Screen-printed carbon electrodes modified with carboxyl functionalized multi-wallet carbon nanotubes	Electrochemical	Environmental application	Phenolics detection			[14]

### 3.4. Other Enzymes

Another commonly used enzyme in biosensors is the tyrosinase, a polyphenol oxidase (Table 4). Tyrosinase is a natural enzyme that may be produced by bacteria, fungi, plants, and mammals. This enzyme catalyzes the oxidation of various phenolics compounds, and their reaction products could be detected by voltamperometric biosensors [60]. The versatility of this enzyme allows it used in the environmental, medical and food field. García-Guzmán et al. [61] developed a biosensor index in beers and wines using caffeic acid as the reference. The biosensor displayed good analytical performance. Additionally, tyrosinase was used in the environmental field for the construction of a biosensor for detection of bisphenol A in water [62]. Alkaline phosphatase (ALP) is extensively used in the diagnosis and monitoring of many diseases. This enzyme catalyzes the dephosphorilation of proteins, biomolecules and nuclei acids. A higher level of ALP is related with tumors, biliary obstruction and diabetes [63]. Moreover, it can be used to detect organophosphate pesticide through catalyzes. Stéfanne e Silva et al. [64] immobilized ALP onto a modified non-commercial, low cost and nonrefundable pencil carbon graphite with three polymers derived from hydroxybenzoic acids for pesticide detection. Urease is an enzyme used for the urea detection, its levels are directly related to the protein intake and nitrogen metabolism in humans. Kim et al. [65] developed a portable biosensor for real time monitoring of the flow of physiological fluids on a porous polytetrafluoethylene. 

## 4. Bi-Enzyme Biosensors

Multi-analyte biosensors can offer the opportunity to perform rapid and cost-effective analysis with a unique sample. Sensitive techniques for multi-analyte detection have become essential in the environment, medical care, food, anti-terrorism, etc. Biosensors for multi-analyte determination do not require complicated and time-consuming procedures and expensive test costs. Additionally, reduction in the physical size of the device is desired, having one electrode with two or more enzymes reduce the space and materials used. Consequently, the biosensors for multianalyte determination are inexpensive. Therefore, several types of research have focused on developing multi-analyte sensors conserving speed, specificity, and sensitivity (Table 5) [71]. Nevertheless, it is essential to find the conditions where all the enzymes have adequate activity and stability and consider that one of the products may have enzyme inactivating effects. Thus, the optimization of the process will be complex because it is necessary to find the conditions where all the enzymes can have good activity for the reaction. Co-immobilization is when two or more enzymes are confined in the same space [72]. It has been proved to be an successful strategy for ordered multi-enzyme immobilization, which can control and enhance the cascade enzymatic reaction rates via adjusting the immobilized sequence [73]. The co-immobilization could use multiple analyte detection of multi-enzymes on an electrode [71]. For example, a bi-enzyme modified electrode with HRP and GOx immobilized by entrapment in poly(noradrenalin) demonstrated to be effective in the monitoring multi-analyte (H_2_O_2_, Cr (III), glucose, and Cr (VI)). This biosensor demonstrates high sensitivity, low LOD, and good selectivity to detect the four analytes [72]. Yokus et al. [74] develop a multiple analyte detection for glucose and lactose. This system had a comparable performance and could quantify and discriminate between two metabolic biomarkers present in sweat. 

**Figure 4:**
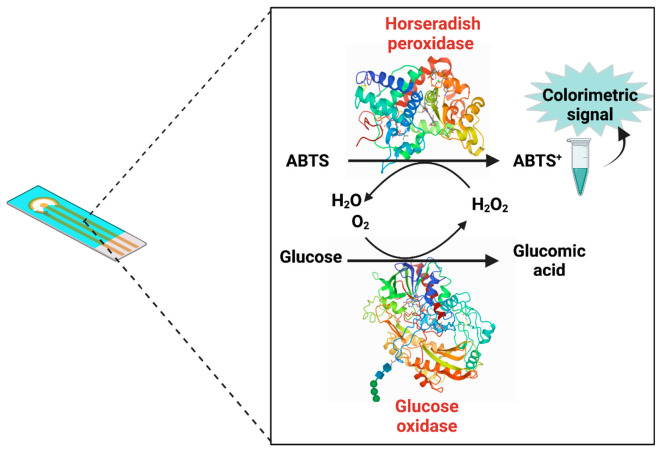
Schematic representation of a bi-enzymatic biosensor.

Multi-enzymatic reactions or cascade reactions occurs in a biosensor with two or more enzymes [75]. The simultaneous use of enzymes has positive effects on reaction performance in cascade reactions. In this process, the product of one enzyme is the other enzyme substrate, as shown in Figure 4. Additionally, co-immobilized enzymes could accelerate initial reaction [72] and enhance the sensitivity of enzyme-biosensors. For example, in the case of glucose biosensors with GOx, it has been shown that an alternative to increasing the biosensor performance (low sensitivity and eliminate interference problems) is the construction of bi-enzymatic peroxidase/oxidase biosensors. In this system H_2_O_2_ generated for glucose oxidation is reduced by HRP. Additionally, it has been proved the enhancement of sensitivity and avoidance. This system has been proved to enhance the sensitivity and prevention of H_2_O_2_ accumulation, which avoids the inactivation of GOx [76]. Additionally, the addition of carbon nanotubes improved the efficiency of biosensors because they facilitated electron transfer. HRP and GOx were co-assembled onto carbon nanotubes modified glass carbon electrodes. HRP provided a biocompatibility microenvironment for the GOx, and the carbon nanotubes facilitated electron transfer. As a result, the biosensor detected glucose based on the consumption of O_2_. Due to the supporting matrix and the cooperation of both enzymes, electrochemical detection of glucose could be achieved with low LOD [77].

**Table 5:** Bi-enzyme systems for biosensors, specifications, and applications.

Enzymes	Transduction System	Material	Application	Detection Range with a Linear Correlation	Limit of Detection (LOD)	Ref.
Glucose oxidase and horseradish peroxidase	Electrochemical	Carbon nanotubes modified glassy carbon electrode	Glucose detection	0.022 to 7.0 mM (R = 0.998)	7 μM	[77]
Glucose oxidase and horseradish peroxidase		Polynoradrenalin/Polyaniline electrode	Glucose	0.50 μM–0.42 mM	0.08 μM	[71]
	Electrochemical		Cr (III)	0.01–3.8 µM		
			Cr (VI))	0.50–6.0 nM	0.20 nM	
HRP and lactate oxidase	Electrochemical	Electrochemical lactate biosensor	Determination of lactate	30.4 μM −243.9 μM	22.6 µM	[78]
Laccase and tyrosinase		Graphite screen printed electrode modified with ferrocene	Phenol	(R^2^ = 0.9994)	2 μM	[79]
	Electrochemical		Gallic acid	(R^2^ = 0.9977)	50 μM	
			Caffeic acid	(R^2^ = 0.9992)	24 μM	
			Catechin	(R^2^ = 0.9930)	40 μM	
Alcohol oxidase and horseradish peroxidase	Electrochemical	Carbon nanotube matrix	Methyl salicylate determination in plants		22.95 μM and 0.98 μM	[80]
D-amino acid oxidase and horseradish peroxidase	Electrochemical	Multi-walled carbon nanotubes and gold nanoparticles modified screen-printed electrode	The total content of D-amino acids	0.020 to 2.0 mM (R = 0.994)	18 μm	[81]
d-amino acid oxidase and hemoglobin	Electrochemical	MnO_2_ nanoparticles enriched poly thiophene	Dopamine	0.04–9.0 μM (R^2^ = 0.994)	12.801 μA/μM and 41 nM	[82]
Cholesterol oxidase and horseradish peroxidase	Electrochemical	Poly(thionine)-modified glassy carbon electrode	Cholesterol	25–125 μM (R = 0.99)	6.3 μM	[83]
Acetylcholinesterase and choline oxidase	Optical	Gold nanorods	Dichlorvos	0.1 to 500 μg/L (R^2^ = 0.963)	8.1 × 10^−3^ μg/L	[84]
			Demeton	1to 500 μg/L (R^2^ = 0.963)	0.32 μg/L	
Glucose oxidase and lactate oxidase	Electrochemical	Flexible electrode array with gold nanoparticles and Prussian blue	Glucose and lactate detection	60 μM-1000 μM (glucose) 5 mM–20 mM (lactate)		[74]
Urease and penicillinase	Electrochemical	Ta_2_O_5_	Urea and penicillin detection	1 mM–25 mM (urea) 0.1 mM–5 mM (penicillin)		[85]

## 5. Nanozyme Biosensors

**Figure 5:**
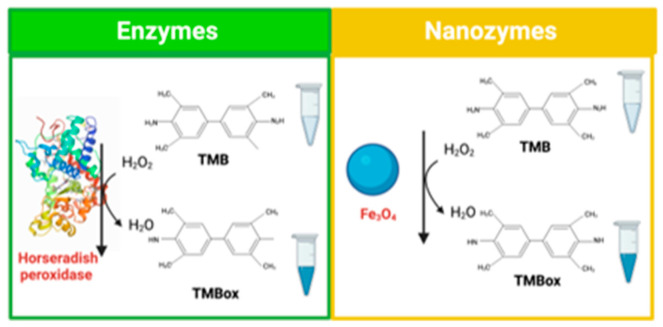
Catalytic action of an enzyme and nanozyme.

Even when enzymatic reactions are highly effective and selective, natural enzymes are difficult to obtain in large quantities, and their catalytic activity is affected by the external environment. Therefore, the study of alternatives to solve the weaknesses of natural enzymes has increased [86]. Nanozymes are nanomaterials that possess unique physicochemical properties and mimic natural enzymes properties (Figure 5) [87]. Significant progress has been made since the report of Zn^2+−^triazacyclonane-functionalized gold nanoparticles with intrinsic peroxidase-like activity due to the rapid development of nanomaterials [88]. In addition, nanozymes offer high structural durability, stability, compatibility with biological materials, remarkable catalytic activity, and material variety. As a result, they are widely used in biosensors for medical diagnosis and environmental monitoring, and they have a huge potential because they are fast, sensitive, efficient, and cheap [89]. Additionally, another exciting advantage of nanozymes is their size/composition-dependent activity. This makes the design of materials with a wide range of catalytic activity possible only by changing the shape, structure, and composition. Besides, their self-assembling ability makes it easier to incorporate biological components into the structure [17]. However, to develop a comparison with the biosensors analyzed before, this section will focus on the nanozymes that mimic the activity of peroxidase, glucose oxidase, and laccase (Table 6) [90,91,92,93,94,95,96,97,98,99,100,101].

Since the discovery of the enzyme-like properties of nanomaterials, several nanomaterials have been employed for the synthesis of nanozymes such as metal oxides (Fe_2_O_3_, NiCo_2_O_4_, and Co_3_O_4_), metal nanoparticles (Ag, Au, Pt, and Pd), metal sulfides (CuS, FeS, and MoS_2_), carbon nanomaterials polymer-coated nanoparticles and nanocomposites [25]. Some materials even have been shown to possess more than one enzyme activity. For example, molybdenum disulfide-based materials (MoS_2_) have peroxidase-like activity, catalase-like activity, and superoxide dismutase activity. Additionally, MoS_2_ is considered a promising material due to its multiple advantages such as simple preparation, low cost, low toxicity, biodegradability, and rapid excretion [102]. Due to nanozymes not having an active site like natural enzymes, different strategies have been made to improve the catalytic properties of these nanomaterials. It has been reported that size, morphology, surface modification composition, pH, and temperature can affect the catalytic performance of nanozymes [103]. Metal nanoparticles (NPs) have the most abundant redox sites, which are considered a great potential for detecting of analytes [104]. Nanozymes have been proved to be used as a potential chemical sensor and biosensor for the detection of glucose, phenols, H_2_O_2_, pesticides, bacteria, cancer cells, among others applications [105]. An official classification of nanozymes has not been established yet [98,103].

### 5.1. Peroxidase-like Activity

The research on nanomaterials with peroxidase-like activity has been growing since the discovery of Fe_3_O_4_ nanoparticles [106,107]. Nanomaterials with peroxidase-like activity reported are metal-based nanoparticles, metal oxide-based nanomaterials, carbon-based nanomaterials (CDs), metal-organic frameworks (MOFs), hybrid nanostructures, among others. These micro and nano-materials possess excellent catalytic properties in colorimetric sensors, pollutants degradation, and disinfection [102,108,109,110]. 

Fe_3_O_4_ nanoparticles had an excellent peroxide-like activity, making it difficult to disperse them in an aqueous solution. To improve the stability to sense the color change, it is necessary to avoid aggregation in water samples. The most common solution is the coating of nanoparticles with compounds that contain functional groups. Christus et al. [90] designed a colorimetric sensor for Hg detection using Fe_3_O_4_ coated with ZnO. They improve the efficiency and selectivity of nanozymes. Another advantage of Fe_3_O_4_ is its comparable catalytic efficiency to HRP, because of the large ferric and ferrous iron area available on their surface. However, Fe_3_O_4_ shows a higher kM for H_2_O_2_ than HRP. The enzyme may have additional contributions to catalysis by its natural active site as amino acid residues. The addition of amino acid residues to the structure of nanoparticles increased the affinity for the substrate. Niu et al. [93] added cysteine residues to the Fe_3_O_4_ nanozyme. Nevertheless, due to the interaction of Cys-Fe, the active site on Fe_3_O_4_ was blocked, and the nanozyme exhibited almost no peroxidase-like activity. Therefore, they used the nanoparticles to create a colorimetric Hg^+2^ sensor. In the presence of Hg^+2^, Cys could be despoiled from the Cys- Fe_3_O_4_ particles by stronger Cys-Hg^2+^-Cys coordination, resulting in the exposure of active Fe_3_O_4_ that could catalyze the oxidation of the substrate, which was the indicator of the Hg^+2^ presence. CDs have some advantages as biocompatibility, low toxicity, tunable luminescent properties, higher water solubility, inexpensive synthesis process [107]. Bimetallic nanozymes can be an option to decrease the cost of the materials and improve their catalytic properties. Nanosheet-structured Cu-CuFe_3_O_4_ has been reported to have higher activity than HRP; however, the affinity to TMB was weaker than HRP.

### 5.2. Oxidase-like Activity

Oxidase-like nanozymes can catalyze the oxidation of substrates to corresponding oxidized products in the presence of O_2_ without H_2_O_2_; unlike peroxidase-like nanozymes, this makes oxidase mimics appropriate for sensing assays with easy operation and high sensitivity. Recently, many inorganic nanomaterials have been found to catalyze the oxidation of substrates exhibiting oxidase-like activity such as Ce, noble metal, Mn, Fe, Cu, Mb. The oxidase-like activity can regulate their physicochemical parameters as size, shape, composition, and surface modification [96,111]. A capping agent can be used to obtain an excellent catalytic activity of nanozyme. He et al. [95] used heparin sodium as a capping agent for obtaining heparin sodium stabilized platinum nanoparticles HS-PtNPs. The nanoparticles could catalyze the oxidation of TMB. Due to isoniazid competed with TMB to bind the active site of the HS-PtNPs, a colorimetric method was designed for isoniazid detection. 

Comotti et al. [112] reported that gold nanoparticles could catalyze glucose to generate gluconic acid and HO_2_ in O_2_ presence. Hydrated glucose anion with gold surface atoms could form electron-rich species that could transform electrons from glucose to dioxygen. Detection of glucose with nanozymes with oxidase-like activity has been reported. Due to the disadvantages of glucose oxidase biosensors for glucose detection, research focuses on developing an enzyme-free glucose sensor using nanomaterials (nanowire, nanorods, nanosheets, nanoparticles and nanotubes). It has been reported the colorimetric detection of glucose by nanozymes. Rashtbari et al. [97] reported nanolayered manganese-calcium oxide nanoparticles with oxidase mimic activity. A non-enzymatic strategy for detection with the naked eye, and quantification of glucose by spectrophotometry was reported. 

### 5.3. Laccase-like Activity

Most of the nanozymes reported have peroxidase, oxidase, or catalase-like activity. Laccase mimic is a new sector of nanozyme research that has been growing in recent years. Many efforts have been made to develop organic/inorganic/hybrid materials with laccase-like activity [6,98]. Laccase has a complex structure of the active site and catalytic mechanism. Due to the catalytic activity of laccase from the active site, which contains copper, copper-based nanocomposites have been fabricated to mimic laccase-like activity. Ma et al. [98] reported that Cu-tannic acid inorganic–organic nanohybrids have excellent laccase-like activity. The reductive property of tannic acid with the reduction of Cu+2 to Cu+ is similar to natural laccases. The biosensor obtained for EP detection showed high tolerance to catalytic activity even when the temperature was increased to 85 °C. The detection limit was less than in previous reports, and the linear range of detection was higher 4.5 to 90 µM. Alizadeh et al. [6] synthesized CuO nanorods to mimic laccase activity and obtained a biosensor that could oxidize EP to a colored product with a LOD of 0.31 µM and linear range of 0.6–18 µM. No interference from ascorbic acid, dopamine, and uric acid was observed. Additionally, considering that the catalytic activity of laccase comes from the bridges formed by copper and cysteine-histidine in the active site, Guan et al. [100] constructed a laccase-like catalyst through the co-assembly of L-cysteine with Cu ions. Cu-cysteine nanoleaves possess a system like laccase with superior activity during long-term incubation. The colorimetric method for EP detection had a linear range 9–455 µM·L^−1^ and a lower limit of detection 2.7 µM·L^−1^. This study shows that the addition of cysteine with copper is more similar than only using copper, thus, better results have been obtained.

Cooper-containing complexes have been synthesized with different organic ligands and carbon dots (CDs) [113]. Ligands such as porphyrins, phthalocyanine, and imidazole have been used to mimic laccases. Additionally, nucleotides are highly versatile ligands, and nucleotide-coordinated Cu+2 complexes have laccase-like activity. The coordination of polymers such as Magnetic Cu/nucleotides has shown excellent laccase-like activity [113]. Liang et al. [114] used nucleotides as ligands and obtained a guanosine monophosphate coordinated copper with tremendous laccase-like activity. CDs have been used as skeletons with active copper as an active center. These nanozymes showed better stability and different optimal pH than natural laccases. Equally, they could detect hydroquinone by the oxidation of p-phenylenediamine to produce a color reaction and were used as fluorescent [115]. 

## 6. Nanozymes-Enzymes Pool 

**Figure 6:**
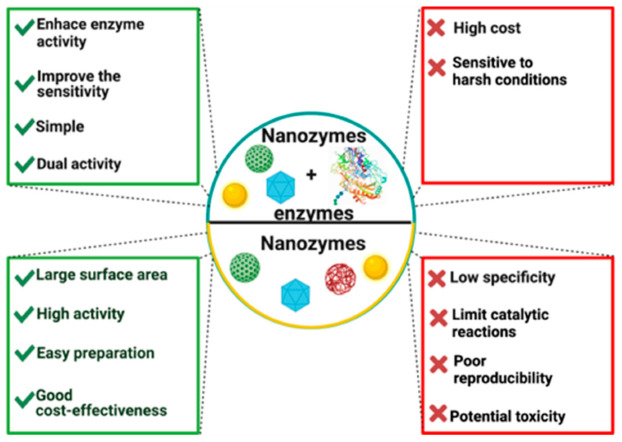
Advantages and disadvantages of nanozymes and nanozymes-enzymes pool.

Enzymatic catalysis is still vulnerable by the poor stability of enzymes. Consequently, studies have focused on the integration of different functional natural or artificial enzyme catalysts. In these systems, enzyme catalysis processes can be promoted by highly stable nanomaterials [116]. As it was mentioned in the previous section, different nanomaterials have been shown enzymatic-like properties. However, even when nanozymes represent a vast potential research potential, some nanozymes still need natural enzymes to detect some molecules as glucose, ethanol, among others (Table 7). Cascade reactions with enzymes are performed for the quantification of the analytes. Helping from the combination of enzymes and nanozymes, the stability of the enzyme can be enhanced and the activity of cascade reactions. Nevertheless, the performance of these systems is affected specialty for their interactions and their kinetic coincidence. Consequently, rational engineering of the multienzyme system architecture is the key to effective cascade reactions and high stability between the two systems [117]. In Figure 6, advantages and disadvantages of nanozymes and nanozymes-enzymes pool are shown. 

Detection of organophosphate pesticides is an example of cascade reaction with the intrinsic peroxidase-like activity of graphene oxide to produce a color reaction in the presence of acetylcholinesterase (AChE) and choline oxidase (CHO) [118]. Similarly, since the quantification of H_2_O_2_ can connect glucose oxidation and TMB oxidation, the nanozymes can be used for the colorimetric detection of glucose, using a chemo-enzymatic cascade system. Tran et al. [119] developed a colorimetric detection of glucose by the combining graphene oxide sheets with silver nanoparticles (AgNPs@rGO) with GOx. GOx catalyzes the glucose oxidation to O_2_, leading to gluconic acid and H_2_O_2_, and H_2_O_2_ can be detected as a product by the nanozyme. Guo et al. [120] synthesized a nanocomposite of cobalt oxide (CoO) with a peroxidase-like activity, assembled onto ordered-mesoporous carbon (CoO-OMC). The nanomaterial shows a good activity upon the oxidation of TMB by H_2_O_2_ to produce a color change, and when GOx was coupled, it was possible to develop a glucose sensor. Smutok et al. [121] developed a sensor for the detection of ethanol and glucose. They synthesized a micro/nanocomposite with peroxidase-like activity based on carbon microfibers modified by hemin and gold nanoparticles coupled with alcohol oxidase and glucose oxidase. They used enzymes to enhance the potential of their biosensor. Parl et al. [108] reported developing a sensor for the colorimetric and fluorometric detection of glucose using a composite metal with platinum and ruthenium (Pt-Ru) by cascade reaction with the glucose oxidase.

Nanozymes are also considered a platform for enzyme immobilization due to nanozymes coupled with natural enzymes that can eliminate the diffusion limitation for substrates and enhance enzymatic activity. Recently, metal-organic frameworks (MOFs) have gained considered attention due to their high potential as a universal platform for the immobilization of enzymes and nanozymes. High flexibility and tunability permit the encapsulation of catalysts with diverse sizes and functions for effective cascade reaction [116].

**Table 7:** Nanozyme-enzyme pool in biosensors.

Enzyme-like Activity	Nanozyme	Enzyme	Transduction System	Application	Range	Limit of Detection	Ref.
Peroxidase-like activity	Carbon microfibers modified by hemin and gold nanoparticles	Alcohol oxidase and glucose oxidase	Electrochemical	Detection of ethanol	0.01–0.15 mM	0.005 mM	[121]
				Detection of glucose	0.1–0.9 mM	0.05 mM	
	Ceria nanomaterials	Glucose oxidase	Optical	Detection of H_2_O_2_	10 μM–50 mM	2 μM	[122]
	Mn (II)/CeO2 nanorods nanocomposites			Detection of glucose	10 μM–100 mM	8.6 μM	
	Silver nanoparticles decorated on reduced graphene oxide sheets (AgNPs@rGO) nanocomposite	Glucose oxidase	Optical	Colorimetric glucose biosensor colorimetric glucose biosensor	125 μM to 1 mM	40 μM	[119]
	Graphene oxide	Acetylcholinesterase and choline oxidase	Optical	Colorimetric detection of organophosphourus pesticides	1–200 ng/mL	2 ppb	[118]
Peroxidase-like activity	Cobalt oxide supported ordered mesoporous carbon (CoO-OMC)	Glucose oxidase	Optical	Colorimetric detection of glucose	0.1–5.0 mM	68 μM	[120]
	Bimetallic PtRu nanoparticles (nPtRu)	Alcohol oxidase and methylamine oxidase	Electrochemical	Food analysis ethanol detection	25–200 µM	3 µM	[123]
				Methylamine detection	20–600 µM	2.5 µM	
	Metallic cobalt nanoparticles encapsulated in metal–organic frameworks derived carbon	Glucose oxidase	Optical	Colorimetric detection of glucose	0.25 to 30 μM	156 nM	[104]
	Prussian Blue	Lactate oxidase	Electrochemical	Detection of lactate			[124]
Peroxidase-like activity	Au nanoparticle/polyluminol	Glucose oxidase	Optical	Detection of glucose	10–1000 μM	10 μM	[125]
	Pt-Ru nanozymes	Glucose oxidase	Optical	Colorimetric and fluorometric glucose detection	0.25–3.0 mM	0.988 and 138 μM	[108]

## 7. Conclusions and Future Perspectives

This review tried to collect some recent trends in enzyme-based biosensors and nanozyme-based biosensors; however, only a small part of the known nanozymes and enzymes used in biosensors is presented in this review. Enzyme-based biosensors and nanozyme-based biosensors have ultrasensitive detection limits and multiple health, food, and environmental applications. In enzyme-based biosensors, horseradish peroxidase, glucose oxidase, and laccase are some of the main reported enzymes. Even when these systems are already commercial products in different applications fields, there is a need to keep improving these technologies. The main challenging issue in the enzyme-based biosensor is the immobilization process due to their stability, shelf life, and reusability related to the efficiency of the immobilization between the platform and the enzyme. Bi-enzyme biosensors represent an excellent alternative for the detection of one or more analytes. The selection of enzymes is an important factor in the development of the biosensor because they must have similar operating conditions (temperature, pH, concentration). 

Using cost-effective nanozymes is a promising way for biosensor development. Nanozyme is progressing faster, that it is difficult to describe all the advances in one review. However, even with all the advantages of using nanozymes, multiple limitations in their application need to be solved, such as the lack of substrate specificity, the fouling of the nanozyme surface due to the absorption of some compounds, and the limited types of enzymes that they can mimic. Therefore, it is required to keep researching the natural active site of enzymes to mimic and enhance the specificity. Additionally, the combination or the synergetic mechanism reported with enzymes and nanozymes represent a promising alternative to face this problem because their interaction could enhance the selectivity and sensitivity of these systems. Future work should focus on understanding the mechanism of interaction between the nanomaterials and enzymes, and on the fabrication of new materials with more enzyme-like activities that could be applied in clinical diagnosis, food analysis and environmental monitoring. 

## Figures and Tables

**Table 2:** Glucose oxidase biosensors.

Material	Transduction System	Application		Linear Range with a Lineal Correlation	Limit of Detection (LOD)	Ref.
ZnO nanorods with chitosan	Electrochemical	Health	Glucose determination	10 μM to 40 μM (R = 0.9998)		[44]
Multi-walled carbon nanotubes and osmium redox polymer	Electrochemical	Health	Glucose determination			[46]
Au nanoparticles (AuNPs) and polynorepinephrine (PNE)	Electrochemical	Health	Glucose determination in human blood serum samples	0.003 mM to 3.43 mM (R^2^ = 0.9987)	1.34 μM	[9]
Glassy carbon electrode with blend nanofibers of poly (vinyl alcohol) and poly(ethyleneimine)	Electrochemical	Health	Glucose in real samples	10 to 30 mmol L^−1^ (R^2^ = 0.971)	0.3 mmol L^−1^	[16]
Screen-printed carbon electrode with platinum nanoparticles electrodeposited on Poly(Azure A)	Electrochemical	Food	Glucose quantification in real samples	20 μM–2.3mM	7.6 μM	[47]

**Table 4:** Other enzymes commonly used in biosensors.

Enzyme	Transduction System	Application		Linear Range with a Lineal Correlation	Limit of Detection (LOD)	Ref.
Lipase	Electrochemical	Environmental application	Methyl parathion detection	0.1–38 μM	0.067 μM	[66]
	Optical	Health application	Triglycerides detection	100–400 mg/dL	15 mg/dL	[67]
Urease	Electrochemical	Health application	Urea detection	1.2–20 mM	1.1 mM	[65]
Tyrosinase	Electrochemical	Environmental application	Bisphenol detection	0.05–20 μM	0.011 μM	[68]
	Electrochemical	Food applications	Caffeic acid (reference polyphenol indices in beers and wines)	10–300 μM	4.33 μM	[61]
	Electrochemical	Environmental applications	Bisphenol detection	5 × 10^−8^–2 × 10^−6^ mol L^−1^	12 nM L^−1^	[62]
	Electrochemical	Food applications	Benzoic acid detection		0.4 μmol L^−1^	[69]
Lactate dehydrogenase	Electrochemical	Health applications	Pyruvate detection	5 × 10^3^–1.4 × 10^5^ nM	8.69 nM	[70]
Alkaline phosphatase	Electrochemical	Environmental applications	Pesticide detection		20 μM	[64]

**Table 6:** Nanozymes (enzyme-like activity) specifications and applications.

Nanozyme	Transduction System	Material	Application	Detection Range with a Linear Correlation	Limit of Detection	Ref.
Peroxidase-like activity	Optical	Fe_3_O_4_@ZnO	Colorimetric sensor for the detection of Hg (II)	0 to 10 nM (R^2^ = 0.9985)	23 nM	[90]
	Optical	Co_3_O_4_@β-cyclodextrin nanoparticles	Colorimetric sensing of ascorbic acid	10–60 μM	1.09 μM	[91]
	Optical	Flower-like yttrium vanadate (YVO_4_) microstructures	Detection of H_2_O_2_	0.5 μM–50 μM	0.126 μM.	[92]
	Optical	Cys-decorated Fe_3_O_4_ nanoparticle	Colorimetric nano-sensor for Hg^2+^ detection (environmental water, human urine and even serum)	0.02–90 nM	5.9 pM	[93]
	Optical	Sodium dodecyl benzene sulfonate (SDBS)-Cu-CuFe_2_O_4_	Detection of H_2_O_2_ and dopamine	0 to 10 μM (R^2^=0.994)	0.32 μM	[94]
Oxidase-like activity	Optical	Heparin sodium and platinum nanoparticles	Pharmaceutical analysis and clinical diagnosis. Colorimetric method for isoniazid	2.5 × 10^−6^ to 2.5 × 10^−4^ M (R^2^ = 0.998)	1.7 × 10^−6^ M	[95]
Oxidase-like activity	Optical	Cerium dioxide nanoparticles	Organophosphorus pesticides	50–1000 ng/mL (R^2^ = 0.9933)	7.6 ng/mL	[96]
	Optical	Nanolayered manganese-calcium oxide nanoparticles	Detection of glucose in real samples	0.0183–0.421 mM	23.86 μM	[97]
Laccase like activity	Optical	Cu-tannic and acid nanohybrids	Colorimetric detection of epinephrine	4.5 to 90 μM (R^2^ = 0.9989)	3.4 μM	[98]
		Coral-like silver citrate microstructures	Catechol	1.87–298 μM	1.03 μM	[49]
			Hydroquinone	2.35–714 μM	1.33 μM	
			2-aminophenol	0.938–714 μM	343 μM	
			2-nitrophenol	7.14–1330 μM	3.15 μM	
			1-naphthol	7.14–579 μM	3.15 μM	
	Optical		2,6-dimethoxyphenol	1.33–298 μM	714 nM	
			4-chlorophenol	0.623–238 μM	343 nM	
			Phenol	0.623–238 μM	343 nM	
	Optical	CH-Cu (Combining key peptides as metal ligands with metal ions)	Detection of epinephrine by a smartphone		0.31 μg/mL	[99]
	Optical and electrochemical	CuO nanorods	Medical diagnosis Colorimetric and electrochemical determination of epinephrine.	0.6–18 μM	0.31 μM	[6]
Laccase like activity	Optical	Copper ion and adenosine monophosphate (AMP-Cu nanozymes)	Detection and remotion of phenolic compounds from fruit juices	0.1–100 μmol·L^−1^	0.033 μmol·L^−1^	[89]
	Optical	Co-assembly of L-cystine with Cu ions	epinephrine detection	9–455 μmol L^–1^	2.7 μmol L^–1^	[100]
Catalase-like and Peroxidase-like dual enzyme mimics	Optical	Ag@Ag_2_WO_4_ NRs	Determination of glucose	27.7 μM to 0.33 mM	2.6 μM	[101]
